# Development trajectory of humanistic care and occupational calling in nursing students during internship: a multi-center longitudinal study

**DOI:** 10.3389/fmed.2025.1632335

**Published:** 2025-10-08

**Authors:** Maoxia Su, Qian Deng, Yi Hu, Yanni Chen

**Affiliations:** ^1^Department of Gastroenterology, Renji Hospital, School of Medicine Chongqing University, Chongqing, China; ^2^The Fifth People's Hospital of Chongqing, Chongqing, China; ^3^Department of Gastroenterology, Chongqing General Hospital, Chongqing, China; ^4^Department of General Surgery, Chongqing Red Cross Hospital, Chongqing, China; ^5^People's Hospital of Jiangbei District, Chongqing, China

**Keywords:** practice nursing students, internship period, humanistic care, professional calling, latent variable growth model, cross-lag model

## Abstract

**Objective:**

To explore the development trajectory of humanistic care and occupational calling of college nursing students and the predictive relationship between them, so as to provide a theoretical basis for improving the occupational calling of nursing students.

**Methods:**

From April 1, 2023 to May 30, 2024, a total of 366 nursing students from 10 colleges and universities in China were selected as the research objects in a longitudinal survey. T1: the early stage of internship (1 to 2 months of internship), T2: the middle stage of internship (4 to 5 months of internship), T3: At the end of internship (8 to 10 months of internship), the humanistic care and occupational calling of nursing students were tracked and investigated, and the cross-lag model and latent growth model were used for data analysis.

**Results:**

342 valid questionnaires were collected (effective recovery rate 93.44%). The cross-lag model showed that: The level of humanistic care at the previous time node significantly positively predicted the occupational calling at the next node (*β* = 0.431, *p* < 0.01, *β* = 0.408, *p* < 0.001), and the occupational calling at the previous time node on average significantly positively predicted the humanistic care at the next node (*β* = 0.426, *p* < 0.001, *β* = 0.414, *p* < 0.001). The latent variable growth model showed that the humanistic care of college nursing students in the internship stage showed an upward trend (*S* = 1.025, *p* < 0.001), and the occupational calling showed an upward trend (*S* = 1.155, *p* < 0.001). At the initial level, humanistic care could predict occupational calling (*β* = 0.458, *p* = 0.002), and both the initial level (*β* = 0.417, *p* = 0.008) and the development speed (*β* = 0.439, *p* < 0.001) of humanistic care could positively predict the development speed of occupational calling. The initial level of occupational calling could positively predict the development speed of occupational calling (*β* = 0.297, *p* = 0.020).

**Conclusion:**

Humanistic care and occupational calling of nursing students are on the rise. College teachers and clinical teachers should pay attention to the evaluation of humanistic care of nursing students, and rationally use the interaction between humanistic care and occupational calling, which has a positive effect on improving nursing students’ occupational calling.

## Introduction

With the vigorous development of medical and health care, the importance of nursing work has become increasingly prominent. From simple disease care to comprehensive services that integrate emotional support, health promotion, and environmental maintenance, the literacy and attitude of nurses have a profound impact on the rehabilitation experience and quality of life of patients (1, 2).

In recent years, “biology - psychology - social medical model” was deepened, the development of nursing science put forward new requirements, need not only solid professional knowledge and skills, need more humanistic concern as a core value concept through every link of nursing practice (3, 4). Nursing humanistic care emphasizes the use of professional knowledge and caring behavior on the basis of respecting the life value of patients to help patients obtain comprehensive rehabilitation in physical (5), psychological and social aspects. For a long time, however, affected by the traditional exam-oriented education concept and pragmatic thoughts and Chinese nursing education in such aspects as curriculum, practice has the tendency to the major than the humanism (6), not only hindered the shape of the humanistic spirit of nursing students, has also weakened their profound cognition and emotion recognition of the value of nursing profession, is not conducive to nursing students to form solid professional beliefs (7).

Occupational calling gives individuals an internal sense of mission and makes them regard career choice as a way to realize their personal ideals and contribute to the society (8), which plays an important role in guiding nursing students’ career identity and career decision-making (9).

In recent years, with the development of positive psychology, more and more studies have begun to explore the strategies and ways to cultivate students’ occupational calling, but the research results specifically for the specific group of college nursing students are still insufficient (10, 11). Positive psychology, by focusing on individual strengths and virtues, provides theoretical support for the humanistic care education of nursing students. Its advocacy of positive emotions, psychological resilience, and the pursuit of meaning can effectively enhance nursing students’ professional identity and sense of value. Humanistic care emphasizes the respect for and fulfillment of nursing students’ emotional and spiritual needs, serving as an important emotional foundation for the formation of their inner vocational calling. As a deep professional motivation, vocational calling arises when nursing students view their work as a meaningful and mission-driven vocation, which requires both positive psychology and humanistic care to jointly nurture their psychological capital and altruistic values.

The humanistic care and occupational calling of nursing students in the internship stage are limited to cross-sectional studies, and the development trajectory and dynamic relationship between them are not clear. Dynamic understanding of the trajectory and interactive relationship between them can better find the inflection point to intervene, so as to improve humanistic care and occupational calling, which is of great significance to improve the professional identity of nursing students and provide better nursing services. This study adopted a longitudinal design, and used the cross-lag model and latent growth model to explore the change trajectory and mutual prediction relationship of humanistic care and occupational calling of nursing students, so as to provide a theoretical basis for university teachers and clinical teachers to improve the occupational calling of nursing students.

## Research objects and methods

### Subjects

This study was a multicenter longitudinal survey. From April 1, 2023 to May 30, 2024, a total of 366 nursing students from 10 colleges and universities in China were selected as the survey subjects. Before the start of this research, submit a written application to the ethics office, a questionnaire survey was conducted after approval. Inclusion criteria: ① Nursing students who completed the learning tasks on campus and entered the clinical practice; ② Informed consent and voluntary participation in this study questionnaire survey. Exclusion criteria: patients who had left nursing practice for more than one month, such as sick leave; the questionnaire was missing more than 1 time. According to the sample size requirement of the latent growth model, at least 200 cases were required, and the sample size was set as *n* = 200/ (1–20%) = 250 due to the high rate of loss to follow-up in the measurement practice stage. A total of 366 nursing students were enrolled in this study.

### Survey instrument

#### General information questionnaire

The general information questionnaire was designed by the researchers, which included general demographic data, the level of hospital where they practiced, and the highest education level.

#### Caring ability inventory (CAI)

The scale was developed by Nkongho and translated into Chinese by Xu (12), a Chinese scholar. A study by Li et al. (13) applied this scale to nursing interns in China, and the results demonstrated that it exhibited high reliability and validity. The scale contained 3 dimensions, namely cognitive dimension, courage dimension and patience dimension, with a total of 37 items. Using Likert7 grading method, from “totally disagree” to “totally agree” were scored from 1 to 7 points, among which 13 items were scored in reverse, and the total score was 37 to 259. The higher the score, the stronger the humanistic care ability. The Cronbach’s *α* coefficients of the scale in this study were 0.870, 0.834 and 0.824.

#### Occupational calling scale

The scale was developed by Dobrow et al. (29) and optimized by Yujing et al. (30) and Shen et al. (14). In this study, the scale was adjusted, for example, “I prefer my current job to other jobs” was adjusted to “I prefer my future nursing job to other jobs.” Li et al. (15) applied this scale to nursing interns, and the results demonstrated that it exhibited high reliability and validity. The scale is a 12-item, single-dimension instrument. Using Likert5 grading method, 1–5 points were scored from “strongly disagree” to “strongly agree,” and the total score was 12–60 points. The higher the score, the stronger the sense of professional calling of nursing students. The Cronbach’s *α* coefficient of the scale was 0.940. The Cronbach’s α coefficients of the scale in this study were 0.885, 0.860 and 0.830.

### Data collection methods

The questionnaire of this study was made by the researcher through wechat Wenjuanxing (ID: 162553770). The questionnaire included the basic information questionnaire and the above two questionnaires. This study followed the voluntary principle, and nursing students voluntarily filled in. The questionnaire was first published on April 1, 2022. The selected time of the longitudinal survey was T1: the beginning of the internship (from January to February), T2: the middle of the internship (from May to June), and T3: the end of the internship (from September to October). All questions are required and must be completed before submission. At the same time, lie detection questions are set to ensure the reliability of the questionnaire.

### Statistical methods

SPSS26.0 and Mplus8.0 software were used for statistical analysis. The count data and measurement data were expressed as the number of cases/percentage and mean ± standard deviation, respectively. Pearson correlation analysis was used for correlation analysis. The missing data were supplemented using the Multiple Imputation (MI) method, and consistency was ensured through sensitivity analysis. The sensitivity analysis demonstrated that the results remained robust after multiple imputation of the missing follow-up data, with the fluctuation of path coefficients being less than 10%. The unconditional linear latent variable growth model (LGM) was used to explore the linear development trajectory of humanistic care/occupational calling of intern nursing students. The intercept of the model represented the initial level of humanistic care/occupational calling, and the slope of the model represented the changing speed of humanistic care/occupational calling. A parallel latent variable growth model was constructed to explore the development trajectory and correlation mechanism of nursing students’ perceptions of caring and occupational calling from a dynamic perspective. The cross-lag model was used to analyze the mutual predictive relationship between humanistic care and occupational calling. Chi-square degree of freedom ratio (χ^2^/df), comparative fit index (CFI), Tucker-Lewis index (Tucker-Lewis index, TLI), root mean square error of approximation (RMSEA), and standardized root mean square residual (SRMR). χ^2^/df < 5.000, CFI > 0.900, TLI > 0.900, RMSEA < 0.080, SRMR < 0.100 indicated a good fit. *p* < 0.05 was considered statistically significant.

## Results

### General demographic data

A total of 342 valid questionnaires were collected with an effective recovery rate of 93.44%. Demographic data of 342 college nursing students, aged 19–27 years old, 38 males (11.11%) and 304 females (88.89%), the specific information is shown in Table 1.

**Table 1:** General information of respondents (*n* = 342).

Items	Categories	*N*	Percentage (%)	Items	Categories	*N*	Percentage (%)
Age (years)	< 20	35	10.23	Level of practice hospital	Grade II and below	15	4.39
	20 ~ 25	280	81.87		3rd Grade B	46	13.45
	> 25	27	7.90		3rd Grade A	281	82.16
Gender	male	38	11.11	Whether he is an only child	Yes	86	25.15
	female	304	88.89		no	256	74.85
Highest degree	College or below	85	24.85	Place of residence	Cities	90	26.32
	Undergraduate	225	65.79		Township	133	38.89
	Master’s degree or above	32	9.36		Rural	119	34.79

### Common method deviation test

There were 11, 10, and 10 factors with characteristic root >1 in T1, T2, and T3 test, respectively. The variation of the three measurements was 16.25, 19.68, and 23.15%, respectively, which were less than the critical value level (16).

Scores and correlation analysis of humanistic care and occupational calling of practice nursing students at three time points.

Pearson correlation analysis was used to analyze humanistic care and occupational calling at three time points, and the results showed that there was a significant correlation between them at three time nodes (*p* < 0.05), which met the prerequisite of cross-lag model and latent variable growth model. The matrix relationship is shown in Table 2.

**Table 2:** Scores and correlation coefficient matrix of humanistic care and occupational calling of practice nursing students at three time points (*r* value, *n* = 342).

Items	Scoring	①	②	③	④	⑤	⑥
① Humanistic care T1	104.23 ± 11.36	1					
② Professional calling T1	35.24 ± 5.20	0.448^**^	1				
③ Humanistic care T2	111.76 ± 12.58	0.563^**^	0.322^*^	1			
④ Professional calling T2	38.38 ± 5.56	0.405^**^	0.479^**^	0.467^**^	1		
⑤ Humanistic care T3	119.46 ± 13.77	0.459^**^	0.303^*^	0.508^**^	0.260^*^	1	
⑥ Professional calling T3	39.95 ± 5.24	0.301^*^	0.368^**^	0.334^**^	0.485^**^	0.425^***^	1

*p < 0.05; **p < 0.01; ***p < 0.001.

### Cross-lag model of humanistic care and occupational calling of practice nursing students

**Figure 1:**
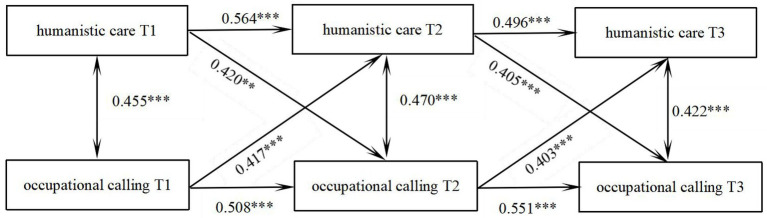
Shows the predictive pathways of humanistic care and occupational calling for trainee nurse at three time points. **p < 0.01; ***p < 0.001.

A cross-lag model was established to investigate the mutual predictive relationship between humanistic care and occupational calling. The model fitted well, χ^2^/df = 0.438, CFI = 0.992, TLI = 0.980, RMSEA = 0.000, SEMR = 0.0346. As shown in Figure 1: The level of humanistic care at the previous time node significantly positively predicted the occupational calling at the next node (*β* = 0.420, *p* < 0.01, *β* = 0.405, *p* < 0.001), and the occupational calling at the previous time node on average significantly positively predicted the humanistic care at the next node (*β* = 0.417, *p* < 0.001, *β* = 0.403, *p* < 0.001). The specific path is shown in Figure 1.

### Humanistic care and occupational calling of nursing students were parallel latent variables

The slope factor loadings of the three longitudinal measurements were set as 0, 1, and 2. The intercept represents the initial level, and the slope represents the development speed.

The development trajectory of humanistic care in practice nursing students.

**Figure 2:**
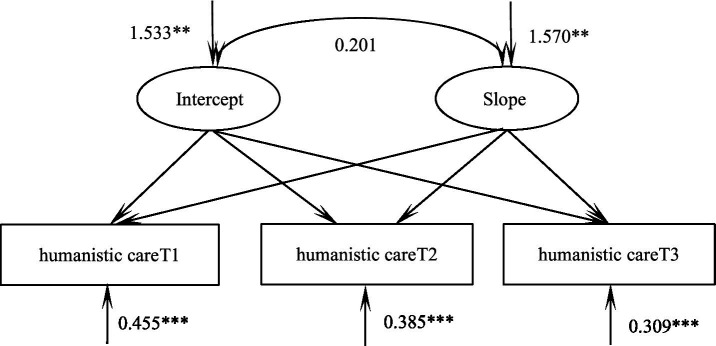
Model of humanistic care for trainee nurse. **p < 0.01; ***p < 0.001.

According to the unconditional latent variable linear growth model of humanistic care of practice nursing students, the fitting indicators were as follows: χ^2^/df = 0.506, GFI = 0.990, TLI = 0.970, RMSEA = 0.056, SRMR = 0.038, and the degree of fitting was good. The intercept of the model was the initial value of humanistic care 105.20, and the subsequent three measurements showed an upward trend (*S* = 1.112, *p* < 0.001). There were significant differences in the variation of intercept (*σ* = ^2^1.533, *p* = 0.005) and slope (*σ*^2^ = 1.570, *p* = 0.003). It shows that there are individual differences in the initial level and development speed of humanistic care among practice nursing students. There was no significant correlation between intercept and slope (*r* = 0.201, *p* = 0.107), suggesting that there was no correlation between the initial level of humanistic care of nursing students and its rising development speed in the later stage (Figure 2).

#### The development trajectory of occupational calling of practice nursing students

**Figure 3:**
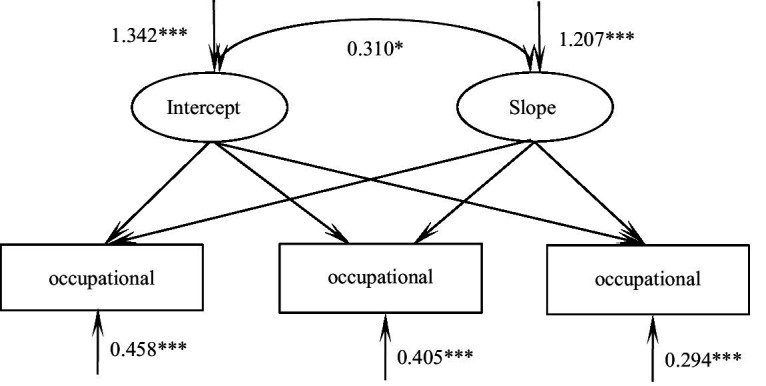
Model of the occupational calling for trainee nurses. *p < 0.05; ***p < 0.001.

According to the unconditional latent variable linear growth model of occupational calling of practice nursing students, the fitting indicators were as follows: χ^2^/df = 2.040, CFI = 0.985, TLI = 0.968, RMSEA = 0.052, SRMR = 0.033, and the degree of fitting was good. The intercept of the model was 35.08, and the following three measurements showed an upward trend (*S* = 1.163, *p* < 0.001). There were significant differences in the variation of intercept (*σ*^2^ = 1.342, *p* < 0.001) and slope (*σ*^2^ = 1.207, *p* < 0.001). These results indicated that there were individual differences in the initial level and development speed of occupational calling among nursing students. There was a significant positive correlation between intercept and slope (*r* = 0.310, *p* = 0.015), suggesting that the higher the initial level of professional calling of nursing students, the faster the later rise rate, Figure 3.

#### To explore the dynamic relationship between humanistic care and occupational calling of nursing students

**Figure 4:**
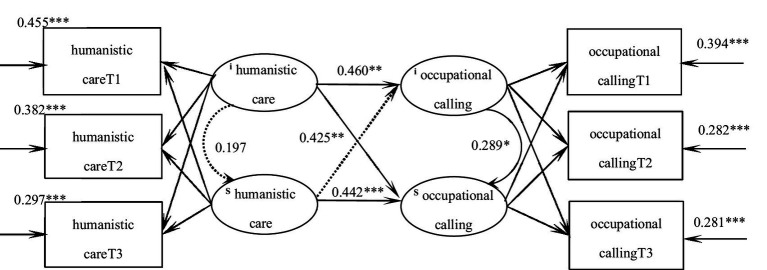
Parellel latent variable model of humanistic care and occupational calling for trainee nursing students. *p < 0.05; **p < 0.01; ***p < 0.001.

The parallel latent variable growth model of humanistic care and occupational calling of practice nursing students was constructed, and the fitting indicators were as follows: χ^2^/df = 2.341, CFI = 0.980, TLI = 0.974, RMSEA = 0.055, SRMR = 0.031, and the degree of fitting was good. At the initial level, humanistic care could predict occupational calling (*β* = 0.60, *p* = 0.004), that is, the higher the initial level of humanistic care of nursing students, the higher the initial level of occupational calling; The initial level of humanistic care could positively predict the development speed of occupational calling (*β* = 0.425, *p* = 0.007), that is, the higher the initial level of humanistic care, the faster the rise of occupational calling. The development speed of humanistic care could positively predict the development speed of occupational calling (*β* = 0.442, *p* < 0.001), that is, the faster the rise of humanistic care in patients, the faster the rise of occupational calling. The initial level of occupational calling can positively predict its development speed (*β* = 0.289, *p* = 0.019), that is, the higher the initial level of occupational calling, the faster its rise. Figure 4 shows the action path.

## Discussion

The changing trajectory of humanistic care and occupational calling of practice nursing students.

Through longitudinal investigation, this study found that humanistic care and occupational calling of nursing students in colleges and universities were on the rise. Clinical practice environment is a key factor for the development of humanistic care and occupational calling of nursing students. Bhuiya et al. (17). found that supportive clinical guidance and a positive team atmosphere could significantly enhance the self-confidence and sense of belonging of nursing students, which prompted them to care more actively for patients. The high level of humanistic care and professionalism shown by experienced nurses and teachers in clinical work will subtly affect college nursing students. In the process of observation and practice, nursing students will internalize these behaviors into their own professional norms and form positive service attitudes and professional attitudes. Therefore, humanistic care showed an upward trend during internship, which was consistent with the previous research results (18). The occupational calling of nursing students shows an upward trend in the internship stage. Internship provides them with the opportunity to experience nursing work and feel the sense of accomplishment of helping patients. At the same time, the guidance and teaching by words and deeds of clinical teachers make them have a deeper understanding of the value of nursing profession. In addition, the experience of caring for patients and solving nursing problems during the internship enhanced their sense of identity and mission to the nursing profession. Nursing students further clarified the essence of nursing. After in-depth understanding of nursing work, they further affirmed their future work, and their sense of professional calling gradually increased.

To explore the relationship between humanistic care and occupational calling of nursing students.

By constructing a cross-lag model, this study found that the level of humanistic care significantly positively predicted the occupational calling at the next node, and the average occupational calling significantly positively predicted the humanistic care at the next node. At the beginning of internship, nursing students actively participate in face-to-face emotional communication with patients, life details care and other behaviors, which is not only respect for patients, but also a sign of the initial formation of professional literacy. Jian et al. (19) pointed out that early humanistic care made nursing students truly feel the value and significance of nursing work, and then initially outlined a positive and clear cognitive outline of the nursing profession, which laid the foundation for the germination of occupational calling. With the progress of internship, nursing students sincerely and continuously provided comprehensive humanistic care to patients, including patient listening to patients’ psychological distress, formulating personalized nursing plans for patients, etc. Humanistic care is not simply the execution of doctor’s advice, but the professional dedication of nursing students with true feelings and feelings. In this process, nursing students gradually touch the soul of the nursing profession and experience the mission of the nursing profession, which gives rise to a high sense of professional identity and further promotes the development of professional calling (20). Therefore, there is a lag in the effect of humanistic care on occupational calling. When nursing students are inspired by strong occupational calling, that is, they are full of vision and enthusiasm for the nursing profession, this passion will drive them to give humanistic care to patients with a more pure and sincere attitude. Hasandoost et al. (21) found through investigation that nursing students with a high level of professional calling would spontaneously regard patients as their own service objects and spiritual care objects, actively increase the frequency of interaction with patients, and not only pay attention to the disease itself, but also give timely encouragement and support to patients when they are depressed. This kind of care from the inside out is more profound and comprehensive. This also shows that there is a lag in the feedback of vocational calling to humanistic care.

Dynamic relationship between humanistic care and occupational calling of practice nursing students.

The results of parallel latent variable growth model showed that at the initial level, humanistic care could predict occupational calling, that is, the higher the level of humanistic care of nursing students, the higher the level of occupational calling. The positive humanistic care behaviors of nursing students during the internship, such as actively communicating with patients, patiently listening to patients’ demands, and providing personalized nursing programs for patients, can significantly enhance their sense of identity with the nursing profession. Lee et al. (22) and Xia et al. (23) pointed out that if nursing students frequently show humanistic care behavior in daily nursing work, they will have a deeper understanding of the value of the nursing profession, and then improve their sense of professional calling. The initial level of humanistic care can positively predict the development speed of occupational calling, that is, the higher the initial level of humanistic care of patients, the faster the rise of occupational calling. In the process of internship, nursing students with higher level of humanistic care are more able to get positive feedback from patients and teachers. These positive evaluations will become an important driving force for the development of nursing students’ career calling, so their career calling development speed is faster (24). The development speed of humanistic care can positively predict the development speed of occupational calling, that is, the faster the rise of humanistic care of patients, the faster the rise of occupational calling. The faster the level of humanistic care of nursing students, the higher the praise of patients or the recognition of teachers, this kind of nursing students have a clearer sense of the significance and sense of achievement of nursing work (25, 26). Frequent positive feedback will strengthen the belief of nursing students to engage in nursing work, and make the level of occupational calling continuously improve (27). The initial level of occupational calling can positively predict its development speed, that is, the higher the initial level of occupational calling, the faster its rise rate. Occupational calling is the emotional identity and sense of mission of nursing profession in the heart of nursing students. Nursing students with a higher initial level of occupational calling are more able to understand the essence of nursing, and are more likely to feel the sense of gain and achievement from clinical work, and then the higher the level of occupational calling in the later stage (28).

### Limitations

Previous research has predominantly focused on cross-sectional investigations of single variables (humanistic care/professional calling), while the humanistic care and professional calling among nursing students during their internship phase exhibit dynamic changes. This longitudinal study investigated the changing trajectories of humanistic care and occupational calling among 342 nursing students throughout their entire internship period, providing a basis for clinical practitioners to dynamically understand their development. Notably, compared to Western cultures that emphasize individualism, Chinese culture places greater importance on collectivism—a cultural characteristic reflected in our findings through higher scores for values like “teamwork” and the observed role of professional identity in promoting “team cohesion.” This suggests that Chinese nurses may be more inclined to derive their sense of professional value from team belonging and harmonious relationships, rather than solely pursuing personal achievement.

However, certain limitations exist. Although it was a multi-center survey, the relatively small sample size may reduce statistical power. Future research will address this issue through large-scale follow-up studies to enhance model stability. Additionally, this study employed a convenience sampling method, and all participants were nursing interns from China. While this offers insights within a collectivist cultural context, it may introduce regional cultural biases and limit the generalizability of the findings. Subsequent studies will include nursing students from multiple countries to optimize the research outcomes and enable cross-cultural comparisons.

## Conclusion

Humanistic care and occupational calling of practice nursing students are on the rise. Humanistic care at the initial level can positively predict occupational calling. Both the initial level and development speed of humanistic care can positively predict the development speed of occupational calling. Clinical educators can demonstrate and guide students in practicing warm communication, empathy, and personalized care during bedside teaching, thereby internalizing humanistic care as a professional habit. Academic educators, on the other hand, need to systematically integrate the concept of humanistic care into curriculum design, such as offering medical ethics courses, communication skills workshops, and employing case analysis and role-playing to help students experience the patient’s perspective. On this basis, both university teachers and clinical instructors should jointly focus on the dynamic evaluation of nursing students’ humanistic care abilities during internships and rationally utilize the interactive and mutually reinforcing relationship between humanistic care and vocational calling. This can be achieved by providing timely positive feedback, systematically recognizing students’ caring behaviors, and organizing reflective discussions to deepen their experiences, thereby effectively strengthening nursing students’ sense of professional meaning and mission, which positively enhances their level of vocational calling and strengthens professional identity.

## Data Availability

The original contributions presented in the study are included in the article/supplementary material, further inquiries can be directed to the corresponding author.
